# Antibody h-R3-dendrimer mediated siRNA has excellent endosomal escape and tumor targeted delivery ability, and represents efficient siPLK1 silencing and inhibition of cell proliferation, migration and invasion

**DOI:** 10.18632/oncotarget.7368

**Published:** 2016-02-13

**Authors:** Jun Li, Jing Liu, Shengnan Li, Yanli Hao, Lei Chen, Xiaoning Zhang

**Affiliations:** ^1^ School of Medicine, Tsinghua University, Beijing 100084, China; ^2^ Collaborative Innovation Center for Biotherapy, Tsinghua University, Beijing 100084, China; ^3^ Department of Gynaecology and Obstetrics, PLA Navy General Hospital, Beijing 100037, China

**Keywords:** siRNA delivery, h-R3, EGFR, siPLK1, targeted delivery

## Abstract

The major obstacle to developing siRNA delivery is their extracellular and intracellular barriers. Herein, a humanized anti-EGFR monoclonal antibody h-R3 was developed to modify the self-assembled binary complexes (dendriplexes) of PAMAM and siRNA via electrostatic interactions, and two common ligands HSA and EGF were used as a control. Compared to dendriplexes, h-R3/EGF/HSA-dendriplexes showed increased particle size, decreased zeta potentials and lower cytotoxicity. Moreover, h-R3-dendriplexes presented greater cellular uptake and excellent endosomal escape ability in HepG2 cells. *Ex vivo* fluorescence imaging revealed that h-R3-dendriplexes showed higher targeted delivery and gene expression in the tumors than dendriplexes, HSA-dendriplexes and EGF-dendriplexes, which was in agreement with confocal results of cryosections. Furthermore, h-R3-dendriplexes for siPLK1 delivery indicated efficient gene silencing, potentiated cell growth inhibition and cell apoptosis, and suppressed cellular migration/invasion. These results indicate that h-R3-dendriplexes represent a great potential to be used as efficient targeted siRNA delivery carriers.

## INTRODUCTION

RNA interference (RNAi) using small interfering RNAs (siRNAs) has recently emerged as a powerful tool for silencing target genes and holds great potential as a novel therapeutic strategy [1–6]. The limitations for siRNA application to clinical success, however, are rapid degradation in the serum, low transfection efficiency, limited internalization into the targeted cell, poor escape from intracellular endosomes and inefficient translocation into the cytoplasm [7–11]. Therefore, efficient and safe delivery of siRNA to the cytoplasm of targeted cells is of great importance to exploit their therapeutic potential [12–14].

Nonviral vectors based on cationic polymers including amine-terminated polyamidoamine (PAMAM) dendrimers as a promising approach have been widely investigated for siRNA delivery [15–18]. These nonviral systems are usually based on electrostatic interactions of negatively charged siRNA with positively charged polymers, resulting in complexes that have a net positive charge [19]. However, this positive charge promotes interaction between the complexes and the negatively charged cellular membrane, resulting in nonspecific adsorptive endocytosis [20]. Moreover, such gene vectors have been investigated to direct efficient siRNA delivery to the liver and lung, but have not been efficient to solid tumors [21]. In addition to the above difficulties, toxicities related with cationic polymers *in vivo* could potentially limit the application of these nonviral vectors [22, 23].

So far, most modification strategies published employ ligands that aid in overcoming delivery barriers, such as eliciting cell surface binding, receptor-mediated endocytosis and avoiding lysosomal degradation to promote delivery to the cytosol [24–29]. Human serum albumin (HSA) and EGF as two common ligands were used to modify the gene therapy carriers. Previous research had indicated that HSA complexed to polyplexes enhances gene silencing for the treatment of breast cancer [30]. Although albumin would not be expected to function as a receptor ligand, it could still facilitate transfection by mediating endocytosis [31, 32]. EGF is a small protein that binds with high affinity to EGF receptor (EGFR), which exerts the promotion of proliferation and differentiation of mesenchymal and epithelial cells. Several works presented that EGF-coated PAMAM complexes significantly increased knockdown of gene expression [33]. However, low transfection efficiency, insufficient cellular uptake and poor targeted delivery *in vivo* still limited its potential for siRNA therapy [34, 35].

To address the limitations of therapeutic siRNA delivery, a new polymeric gene delivery system based on antibody h-R3 and PAMAM, is described that enhances intracellular delivery of siRNA. Nimotuzumab (h-R3) is a humanized monoclonal antibody (mAb) against human epidermal growth factor receptor (EGFR) that demonstrated a remarkable antiproliferative, pro-apoptotic and antiangiogenic effect [36–38]. Unlike other anti-EGFR monoclonal antibody, such as mAbs C225 and ABX-EGF, h-R3 did not provoke acneiform rash or folliculitis [39]. Also, h-R3 represents different pharmacokinetic properties with more prolonged half-life and a higher area under the curve (AUC) at the dose levels associated with systemic clearance saturation [40]. In addition, our previous work has showed that h-R3-mediated delivery system represented higher transfection efficiency of plasmid DNA and targeted delivery in EGFR-overexpressing tumor cells [41].

In this study, self-assembled h-R3/EGF/HSA-PAMAM-siRNA ternary complexes (h-R3/EGF/HSA-dendriplexes) were prepared using electrostatic adsorption of PAMAM-siRNA binary complexes (dendriplexes) with negatively charged ligand (h-R3/EGF/HSA). And, physicochemical properties (including siRNA loading ability, particles size, zeta potential and morphology), *in vitro* toxicity, gene transfection efficacy, intracellular uptake and endosomal escape ability in EGFR-overexpressing HepG2 cells were evaluated. Furthermore, *ex vivo* distribution and gene expression of dendriplexes and h-R3/EGF/HSA-dendriplexes were determined in tumor-bearing BALB/c nude mice. To test the potential of such novel siRNA delivery system in tumor therapy, we further investigated this h-R3-mediated siRNA delivery system, compared with dendriplex, HSA-dendriplex and EGF-dendriplex, in PLK1-siRNA (siPLK1) delivery against HepG2 cells and tested the efficacy, including gene silencing, cell growth inhibition, cell apoptosis and cellular migration/invasion.

## RESULTS AND DISCUSSION

### Formulation of siRNA delivery system

**Figure 1:**
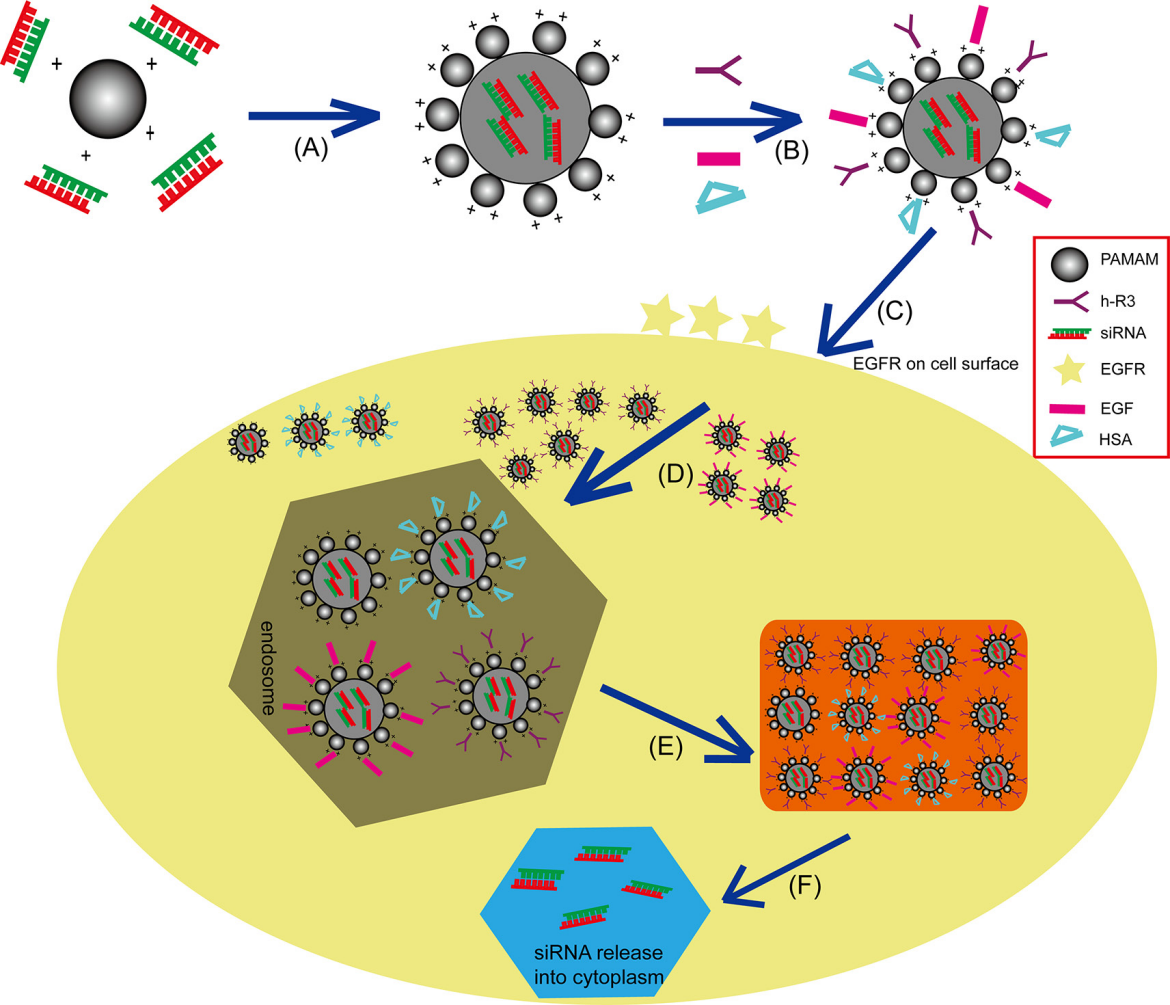
Schematic representation of the siRNA gene delivery system (**A**) Electrostatic interactions of PAMAM and siRNA to form complexes (dendriplexes). (**B**) Self-assembled h-R3/EGF/HSA-dendriplexes via electrostatic adsorption of dendriplexes to negatively charged h-R3/EGF/HSA. (**C**) Specific binding of h-R3/EGF to the EGFR receptors on the HepG2 tumor cell surfaces; (**D**) Receptor-mediated endocytosis and captured by the endosomes. (**E**) Endosomal escape. (**F**) Release of siRNA into cytoplasm.

Cationic PAMAM dendrimers are unique highly branched polymers with surface amino groups that they allow functional modifications to be performed under mild conditions [42]. Recently, these polymers modified with various agents such as PEG, RGD, arginine and cyclodextrin, have been widely investigated as excellent nonviral vectors for siRNA delivery in different tumor models *in vitro* and *in vivo* [43–46]. In this study, the negatively charged anti-EGFR antibody h-R3 was designed to modify the positively charged PAMAM-siRNA binary complexes (dendriplexes), and two another common ligands (HSA and EGF) were used as control. Figure 1 presents the schematic representation of these h-R3/EGF/HSA-PAMAM siRNA delivery systems for tumor therapy. Firstly, self-assembled h-R3/EGF/HSA-dendriplexes via electrostatic adsorption of PAMAM-siRNA complexes (dendriplexes) to negatively charged h-R3/EGF/HSA were designed. Subsequently, more EGF/h-R3-dendriplexes could be uptake with binding of h-R3/EGF to the EGFR receptors on the HepG2 tumor cell surfaces. Then, the complexes internalized into endosomes, however, the proton sponge effect caused by PAMAM dendrimer can trigger endosomal escape. And, importantly, h-R3-dendriplexes had excellent endosomal/lysosomal escape ability. Finally, siRNA separated from complexes and released into cytoplasm.

### Characterization of dendriplexes and ligand-dendriplexes

**Figure 2:**
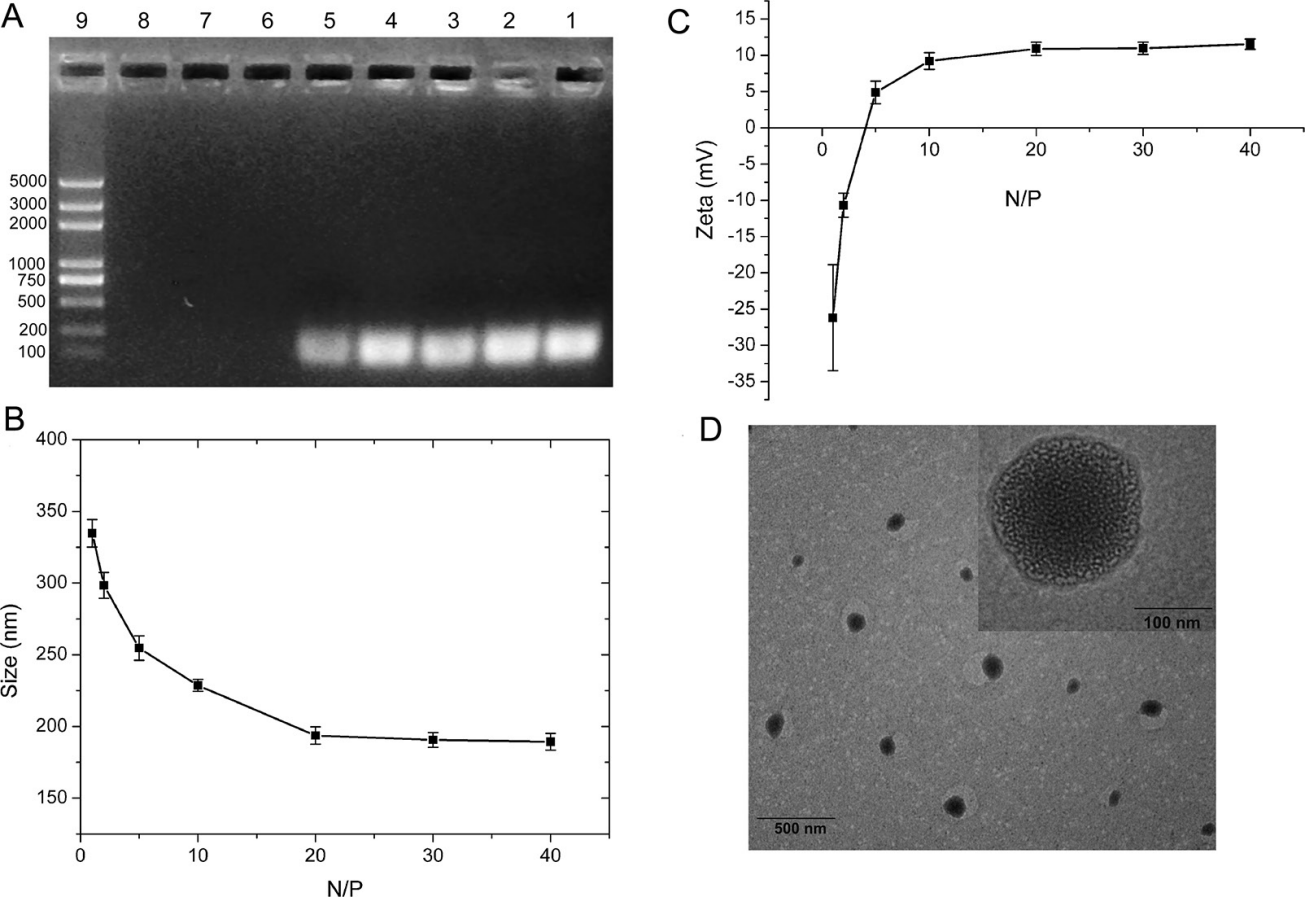
Characterization of dendriplexes (**A**) Agarose gel retardation assay of dendriplexes at different N/P ratio (1, 2, 5, 10, 20, 30, 40). Lane 1: siRNA; Lane 2-8: dendriplexes at N/P ratio of 1, 2, 5, 10, 20, 30, 40; Lane 9: Maker. (**B**) Particle Sizes of dendriplexes at different N/P ratio. Results were expressed as mean ± standard deviation (*n* = 3). (**C**) Zeta potentials of dendriplexes at different N/P ratio. Results were expressed as mean ± standard deviation (*n* = 3). (**D**) TEM image of dendriplexes at N/P ratio of 20. Scale bar is 100 nm.

As shown in Figure 2A, the formulation of PAMAM-siRNA complexes (dendriplexes) with different N/P ratio was assessed by the agarose gel retardation assay. When the N/P ratios were reached 20, the PAMAM dendrimer was able to completely bind siRNA to form dendriplexes. Dynamic light scattering (DLS) was applied to determine the particle size and zeta potentials of dendriplexes. The particle sizes of different complexes were measured as shown in Figure 2B. All sizes of dendriplexes were less than 194 nm when N/P ratios reached 20, which indicated siRNA could be effectively condensed by PAMAM at these N/P ratios. As seen from Figure 2C, zeta potentials of the dendriplexes increased with increasing N/P ratio when the N/P ratio was less than 20. As we know, DLS method can not measuring the real size of nanoparticles (only detect light scattering which can be used to calculate particle sizes), transmission electron microscopy (TEM) as an additional and useful technique can thus provide direct information on the particle size and morphology. Figure 2D shows the TEM images of dendriplexes at N/P ratio of 20. The results reveal spherical structures with mean particle size about 175 nm. However, the particle size from TEM was smaller than that determined by DLS, which could be explained by the collapse of particles during the sample preparation of TEM. Moreover, the particle size results of these siRNA complexes partially met the enhanced permeability and retention (EPR) effect that facilitated to accumulate in tumors [47, 48].

**Figure 3:**
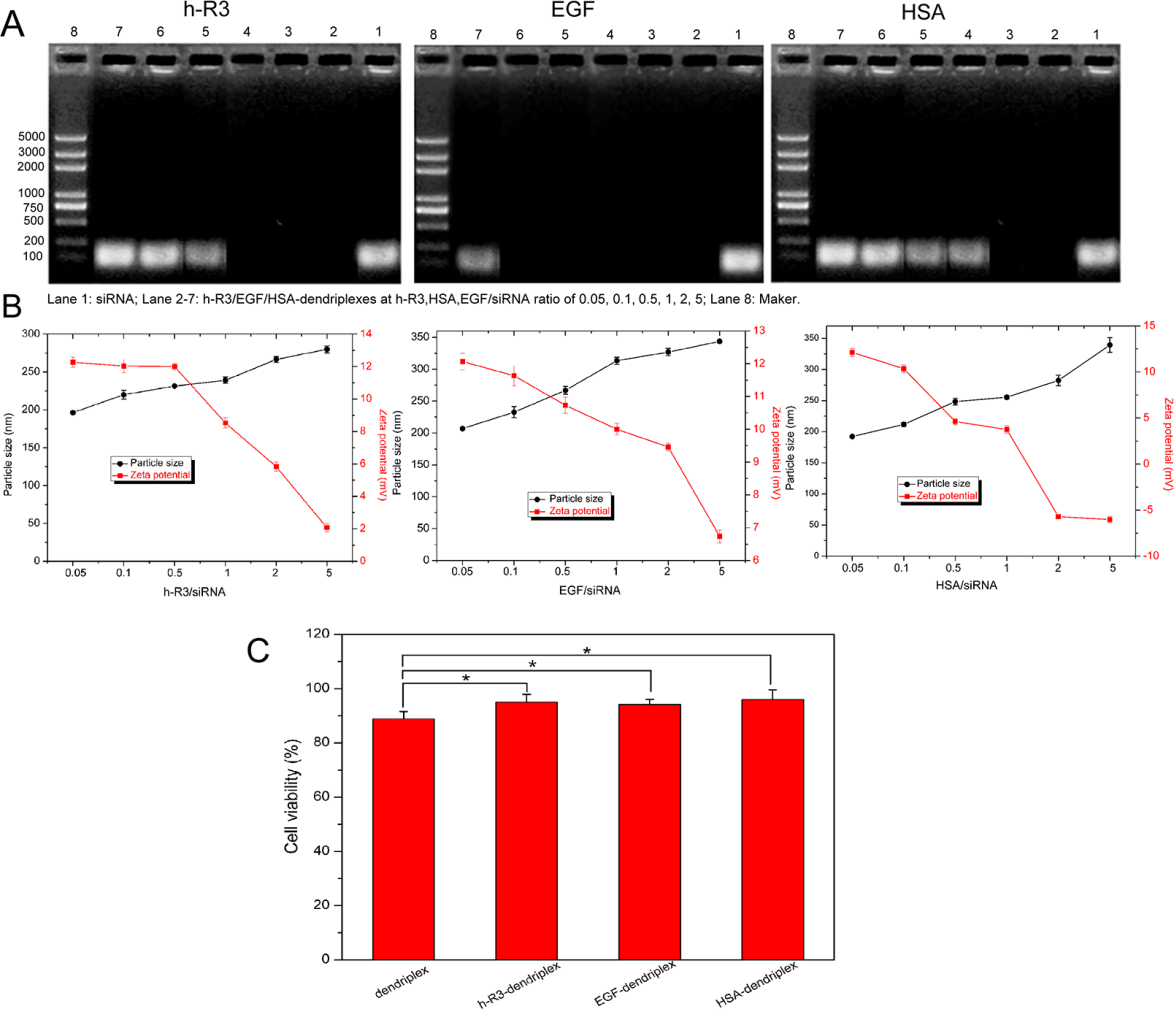
Effect of ligand (h-R3/EGF/HAS) on characterization of dendriplexes (**A**) Effect of ligand on agarose gel retardation assay of dendriplexes. Lane 1: siRNA; Lane 2–7: h-R3/EGF/HSA dendriplexes at h-R3, EGF, HSA/siRNA ratio of 0.05, 0.1, 0.5, 1, 2, 5, Lane 8: Maker. (**B**) Effect of ligand on the particle sizes and zeta potentials of dendriplexes (N/P 20:1; ligand/siRNA ratio of 0.05, 0.1, 0.5, 1, 2, 5). (**C**) Cell viability after transfection mediated by dendriplexes (N/P 20:1) and h-R3/EGF/HSA-dendriplexes (ligand/siRNA 0.5:1) valuated by MTT assay. Error bars indicate s.d. (*n* = 3), *p < 0.05.

The effect of ligands such as h-R3, EGF and HSA on formulation of dendriplexes was detected by agarose gel retardation assay, as shown in Figure 3A. For h-R3-dendriplexes with an N/P ratio of 20, h-R3-dendriplexes could not completely prevent siRNA from migrating into the gel when the weight ratio of h-R3/siRNA was 1, indicating that, in order to bind siRNA completely, the weight ratio of h-R3/siRNA should be less than 1. And, for EGF-dendriplexes and HSA-dendriplex, the weight ratio of EGF/siRNA and HSA/siRNA should be less than 5 and 0.5. This can be attributed to a competition in the interaction between anionic siRNA and negative ligands (h-R3/EGF/HSA) with the positive PAMAM. Figure 3B shows the effect of ligand on the particle sizes and zeta potentials of dendriplexes. When the amount of ligand (h-R3/EGF/HSA) increased, particle size increased and zeta potentials decreased. With the above results, the ligand/siRNA ratio of 0.5 was used for further investigated.

### *In vitro* cytotoxicity

The cytotoxicity of dendriplexes and ligand (h-R3/EGF/HSA)-dendriplexes in HepG2 cells was evaluated by MTT assay. As seen in Figure 3C, compared to dendriplexes, the viability of treated with ligand-dendriplexes was increased relatively (more than 90%), which represented the good biocompatibility of ligand-dendriplexes. In this study, ligand (h-R3/EGF/HSA) was used to partially neutralize the positive charge of dendriplexes. The ligand-dendriplexes showed relatively lower zeta potential as compared with dendriplexes (Figure 2B), which resulted in their reduced cytotoxicity.

### Cellular uptake

**Figure 4:**
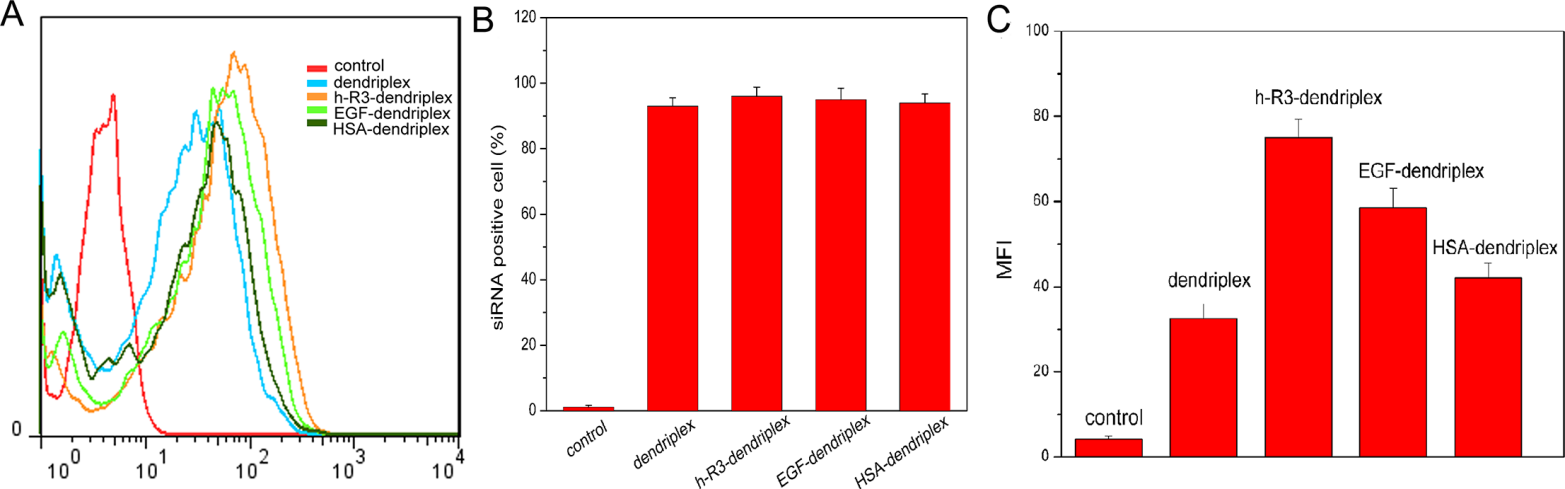
Assessment of cellular uptake using flow cytometry (**A**) Intracellular fluorescence intensities of dendriplexes and h-R3/EGF/HSA-dendriplexes determined by flow cytometry. (**B**) siRNA-positive cells after treated with different Cy5-siRNA formulations. Error bars indicate s.d. (*n* = 3). (**C**) Mean fluorescence intensity (MFI) of dendriplexes and h-R3/EGF/HSA-dendriplexes with Cy5-labeled siRNA measured by flow cytometry. Negative control was the group without any treatment. Error bars indicate s.d. (*n* = 3).

Cy5-labeled siRNA was used as an indicator to analyze the intracellular distribution of dendriplex, h-R3-dendriplex, EGF-dendriplex and HSA-dendriplex in HepG2 cells. After 24 h transfection, intracellular fluorescence intensities (Figure 4A) and the percentage of the cells that internalized Cy5-labeled siRNA (Figure 4B) were evaluated by flow cytometry. Intracellular fluorescence intensity of Cy5-siRNA was in the order of h-R3-dendriplex > EGF-dendriplex > HSA-dendriplex > dendriplex. Compared to the untreated cells (control), the percentages of siRNA-positive cells of h-R3-dendriplex, EGF-dendriplex, HSA-dendriplex and dendriplex were more than 90%, which showed extremely higher uptake efficiencies. However, the mean fluorescence intensity showed to be different (Figure 4C), the h-R3-dendriplex presented the highest mean fluorescence intensity compared to dendriplex, HSA-dendriplex and EGF-dendriplex. It was reported by Liang research group that higher mean fluorescence intensity could be related to higher targeted delivery effectiveness [49].

### Endosomal/lysosomal escaping of siRNA

**Figure 5:**
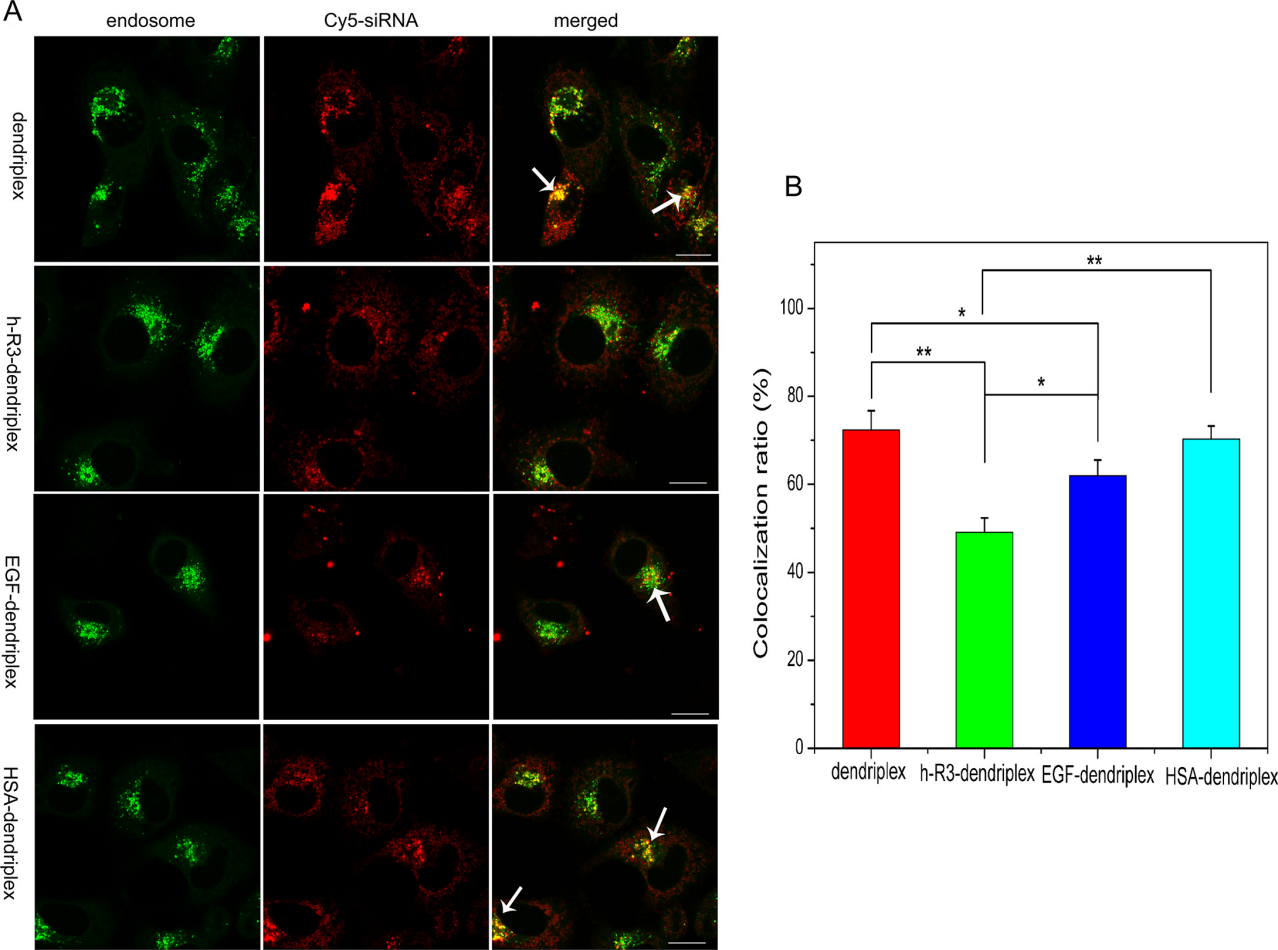
Intracellular localization and endosomal escape observed by confocal laser scanning microscopy (CLSM) (**A**) Intracellular localization of dendriplex and h-R3/EGF/HSA-dendriplex (N/P 20:1, h-R3, EGF, HSA/siRNA 0.5) in HepG2 cells observed by confocal laser scanning microscopy. (Green: LysoTracker Green used to stain endosomes; red: Cy5-labled siRNA; yellow: colocalization). The arrows indicate the co-localization of siRNA with endosomes. Scale bars = 10 μm. (**B**) Colocalization ratio of Cy5-labeled siRNA with LysoTracker (endosomes) calculated by pixel counting (number of counted cells: 5, Error bars indicate s.d. *p < 0.05, **p < 0.01).

The successful escape of siRNA carriers from endosomes is crucial to efficient transfection efficiency, and siRNA should be dissociated from its carriers in the cytoplasm for efficient gene silencing [50]. Confocal microscopy can be used to investigate the intracellular distribution of dendriplexes. Figure 5A shows the intracellular localization of dendriplexes and h-R3/EGF/HSA-dendriplexes in HepG2 cells. In the CLSM images, the colocalization of Cy5-siRNA (red) with endosomes/lysosomes (green) should be observed as yellow. On the other hand, if the Cy5-siRNA is able to escape from the endosomes/lysosomes and release into the cytoplasm, the fluorescence signal will not be colocalized with the LysoTracker green. For the transfection experiment, cells were incubated with dendriplexes and h-R3/EGF/HSA-dendriplexes for 5 h and CLSM images were taken after incubation for 24 h. More obvious yellow regions were observed in unmodified dendriplex and HSA-dendriplex, while fewer yellow colors were found in cells treated with h-R3-dendriplex and EGF-dendriplex. This indicated that more h-R3-dendriplex and EGF-dendriplex were visible in the cells and released into cytoplasm while not in the endosome.

Based on the numbers of red and yellow pixels in the CLSM images, the colocalization ratio of Cy5-siRNA with late endosomes was further estimated. As shown in Figure 5B, the colocalization ratio was highly significantly decreased to 49.1% for the cells treated with h-R3-dendriplex, whereas that for the cells treated with unmodified dendriplex (72.4%), EGF-dendriplex (61.9%) and HSA-dendriplex (70.3%). The results demonstrated that h-R3-dendriplexes were more effective for siRNA to escape from endosomes. Therefore, these results suggest that h-R3 may contribute to facilitate endosomal escape of the dendriplexes and to enhance transfection efficiency.

### *Ex vivo* distribution by fluorescence imaging and confocal microscopy

**Figure 6:**
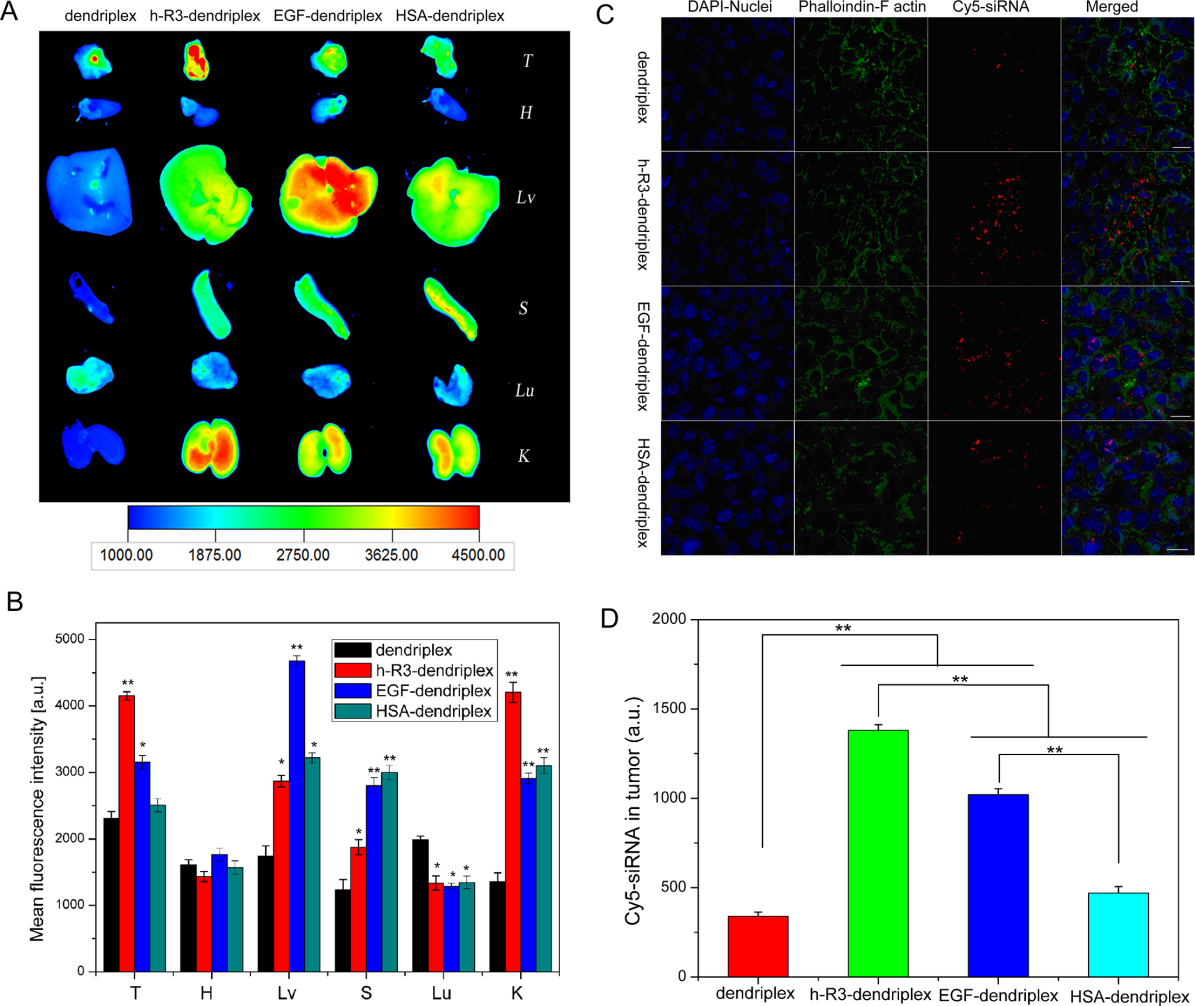
*Ex vivo* distribution and siRNA delivery (**A**) *Ex vivo* fluorescence imaging of five major organs and tumor 24 h post-injection after injection with dendriplex and h-R3/EGF/HSA-dendriplex (T, tumor; H, heart; Lv, liver; S, spleen; Lu, lung; K, kidney). (**B**) Region-of-interest analysis of fluorescent signals from the tumors and five major organs 24 h post-injection. (T, tumor; H, heart; Lv, liver; S, spleen; Lu, lung; K, kidney). Error bars indicate s.d. (*n* = 3). **P < 0.01. *p < 0.05 compared with dendriplexes as the control. (**C**) *Ex vivo* distribution of siRNA in the tumor treated with dendriplex and h-R3/EGF/HSA-dendriplex 24 h after i.v. injection to nude mice-bearing HepG2 tumor. Cryosections of tumor tissues (6 um thick) were examined by CLSM. DAPI and fluorescein isothiocyanate-labeled phalloidin were used to stain nuclei and F actin (to display the rough cell outline), respectively. Red homogeneous spots present the Cy5-siRNA. Scale bars = 50 μm. (**D**) Quantitative analysis of Cy5-siRNA in tumor was executed by Image-Pro Plus. Error bars indicate s.d. (*n* = 3). **P < 0.01.

Fluorescence measurements offer a direct study of the biofate of nanoparticles for drug and gene delivery [51, 52]. In this study, Cy5-labeled siRNA was used to form dendriplexes and h-R3/EGF/HSA-dendriplexes for fluorescence imaging to determine the biodistribution. Figure 6A shows *ex vivo* imaging results of five major organs and tumor 24 h after i.v. injection. Compared to dendriplex, EGF-dendriplex and HSA-dendriplex, h-R3-dendriplexes represented higher accumulation in the tumor. Moreover, the ligand-dendriplexes showed the higher accumulation in liver and spleen, which can be explained by the fact that ligand-dendriplex presented the larger size than unmodified dendriplexes (Figure 3B), because phagocytosis by the Kupffer cells and spleen macrophages are mostly dependent on particle size.

Figure 6B shows the region-of-interest analysis (ROI) of fluorescence intensities from five major organs and tumor. As we know, due to the strong phagocytosis in reticuloendothelial system (RES), nanoparticles are usually accumulated in the liver and spleen with much higher content than other organs and tumor [53]. More interestingly in this study, the fluorescence intensity of h-R3-dendriplex in tumor was approximately 1.5 times than that in liver, and 2.3 times than in spleen. This result revealed that these h-R3-mediated delivery systems represented big advantages in tumor-targeted siRNA delivery. Furthermore, It was reported that siRNA distribution mostly preferred to accumulate in lung [54], however, fluorescence intensity of ligand-dendriplexes in lung decreased in this study. The decreased accumulation in the lungs can be attributed to the effect to ligand (h-R3/EGF/HSA) modification of dendriplex.

To further evaluate the differences between dendriplexes, h-R3-dendriplexes, EGF-dendriplexes and HSA-dendriplexes, we used CLSM to observe the complexes distribution in tumors. In this study, siRNA labeled by Cy5 dye, nuclei stained with DAPI and phalloidin staining F-actin were employed to identify the siRNA localization in tumors. As shown in Figure 6C and 6D, h-R3-dendriplexes exhibited stronger fluorescence signals than dendriplexes, HSA-dendriplexes and EGF-dendriplexes. These CLSM results of cryosections in tumors are in accordance with the *ex vivo* fluorescence imaging results. And, the more notable signals of Cy5 in h-R3-dendriplexes were probably due to the enhanced cellular uptake and endosomal/lysosomal escape capacity as discussed in Figure 5.

### Ligand-dendriplexes for PLK1-siRNA (siPLK1) delivery

**Figure 7:**
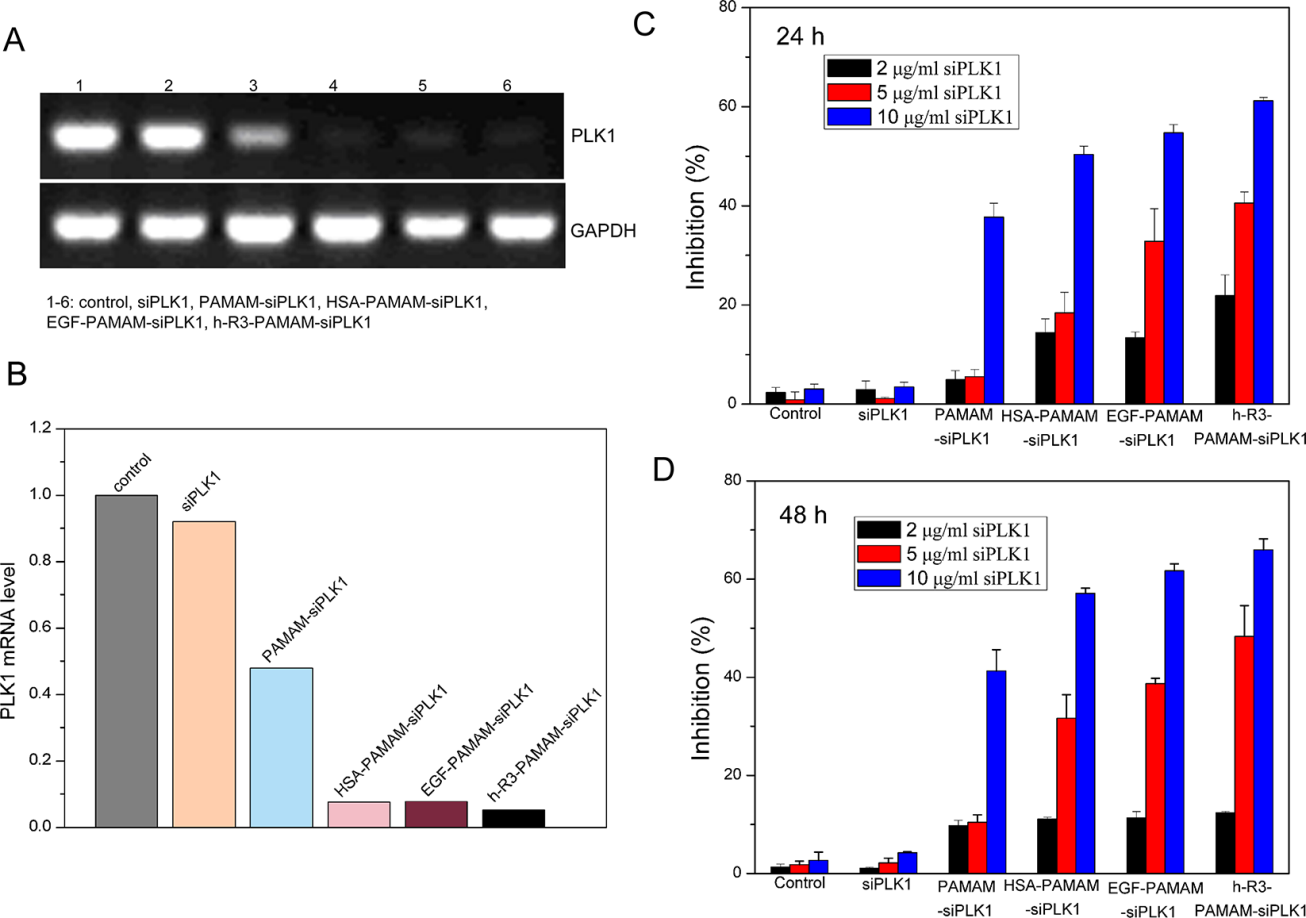
Ligand (h-R3/EGF/HSA)-dendriplexes for siPLK1 delivery (**A**) Expression of PLK1 at mRNA after transfection with different samples (naked siPLK1, PAMAM-siPLK1, HSA-PAMAM-siPLK1, EGF-PAMAM-siPLK1 and h-R3-PAMAM-siPLK1 in HepG2 cells by RT-PCR; N/P 20:1, HSA, EGF, h-R3/siPLK1 0.5:1. (**B**) Quantitative presentation of PLK1 were calculated by Smart View software after normalization with control sample. (**C**) Cell growth inhibition induced by different siPLK1 concentration (2 μg/ml, 5 μg/ml, 10 μg/ml) treated after 24 h. N/P 20:1, HSA, EGF, h-R3/siPLK1 0.5:1. Results were expressed as mean ± standard deviation (*n* = 5). (**D**) Cell growth inhibition induced by different siPLK1 concentration (2 μg/ml, 5 μg/ml, 10 μg/ml) treated after 48 h. N/P 20:1, HSA, EGF, h-R3/siPLK1 0.5:1. Results were expressed as mean ± standard deviation (*n* = 5).

Polo-like kinase-1 (PLK1) belongs to a member of a conserved family of serine/threonine kinases that mediates various mitotic events, and its activity is elevated in a broad range of human tumor cell lines [55, 56]. Silencing PLK1 effectively is most important to siRNA delivery for tumor therapy. Expression of PLK1 at mRNA in HepG2 cells treated with different samples was detected by RT-PCR (Figure 7A). The results represented appreciable alterations in PLK1 mRNA levels after transfection with h-R3-PAMAM-siPLK1, HSA-PAMAM-siPLK1 and EGF- PAMAM-siPLK1 samples compared to naked siPLK1 and PAMAM-siPLK1. Expression of GAPDH was used as an internal control to demonstrate equal loading of RNA samples. Quantitation of data (Figure 7B) demonstrated silencing efficacy reached 94.8% after treatment with h-R3-siPLK1-PAMAM complexes, compared with naked siPLK1 (7.9%), siPLK1-PAMAM (52%), HSA-siPLK1-PAMAM (92.5%) and EGF-siPLK1-PAMAM (92.1%) complexes.

To evaluate cell proliferation, we used MTT assays (Figure 7C and 7D). An efficient inhibition of cell proliferation was observed after treatment with h-R3-siPLK1-PAMAM complexes for 6 h and subsequent incubation of up to 24 h or 48 h, relative to the naked siPLK1, HSA-siPLK1-PAMAM and EGF-siPLK1-PAMAM complexes as well as untreated sample. And, the result also showed that the increased siPLK1 concentration in the complexes led to high tumor cell inhibition.

**Figure 8:**
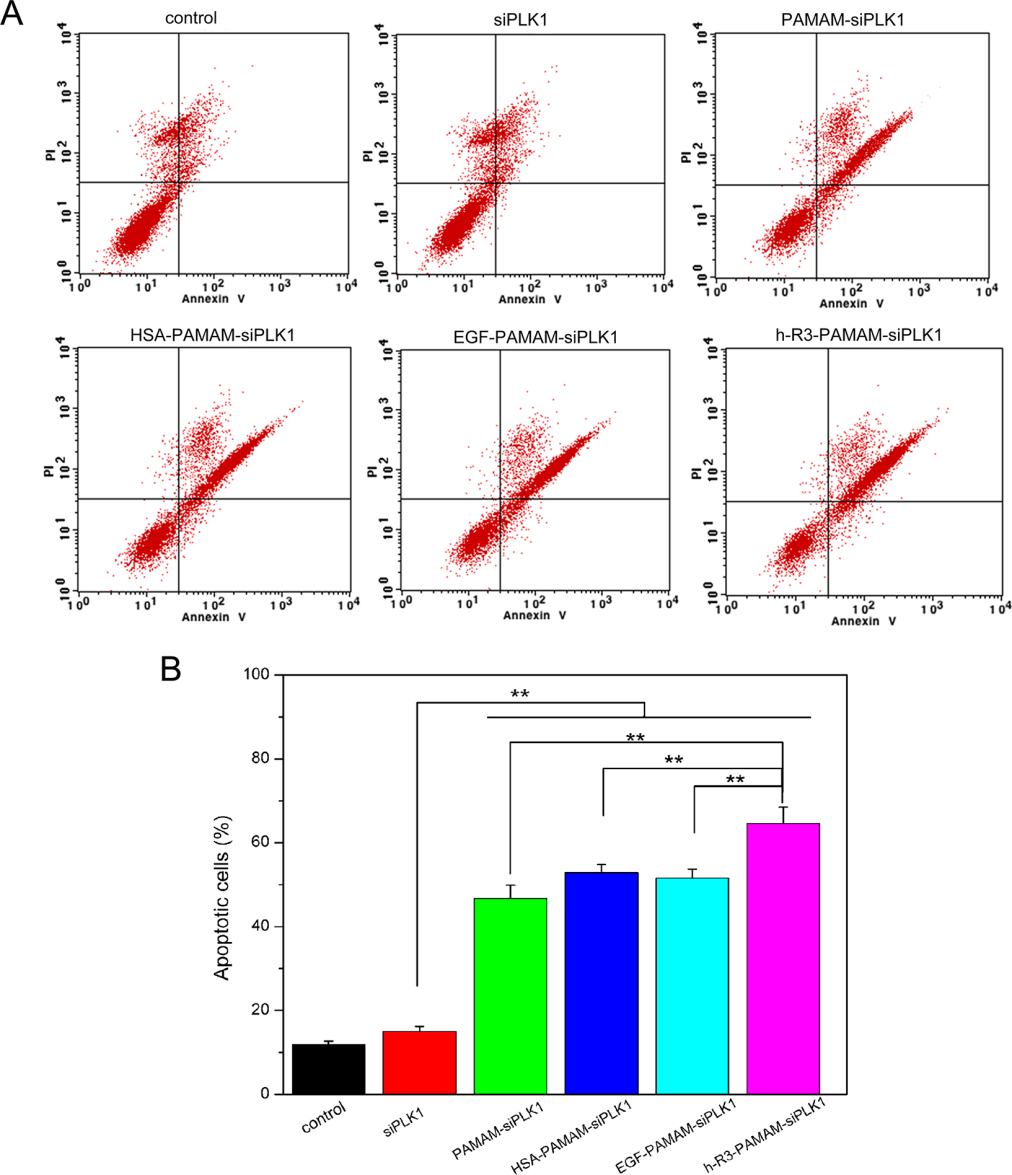
FACS analysis for detection of apoptotic cells (**A**) FACS dot plots of HepG2 cells with different samples (siPLK1, PAMAM-siPLK1, HSA-PAMAM-siPLK1, EGF-PAMAM-siPLK1, h-R3-PAMAM-siPLK1); (**B**) Quantitative presentation of DNA fragmentation data from FACS analysis to indicate percent changes in apoptotic cells. Error bars indicate s.d. (*n* = 3). **P < 0.01.

To assess whether cell apoptosis accounted for the loss of viability, we examined by FACS analysis. The highest cell population in the apoptotic cells area of h-R3-PAMAM-siPLK1 samples was shown in the FACS dot plot graphs (Figure 8A). We presented the quantitative FACS data for percentage of apoptotic cells based on DNA fragmentation (Figure 8B). Quantitation of data demonstrated 15.04%, 47.83%, 51.55%, 49.62% and 63.59% apoptotic cells after treatment with naked siPLK1, siPLK1-PAMAM, HSA-siPLK1-PAMAM, EGF-siPLK1-PAMAM and h-R3-siPLK1-PAMAM complexes (Figure 8B). The h-R3-PAMAM-siPLK1 with an increase in cell apoptosis compared to treatment with other samples could make cause to cell inhibition (Figure 7C and 7D).

**Figure 9:**
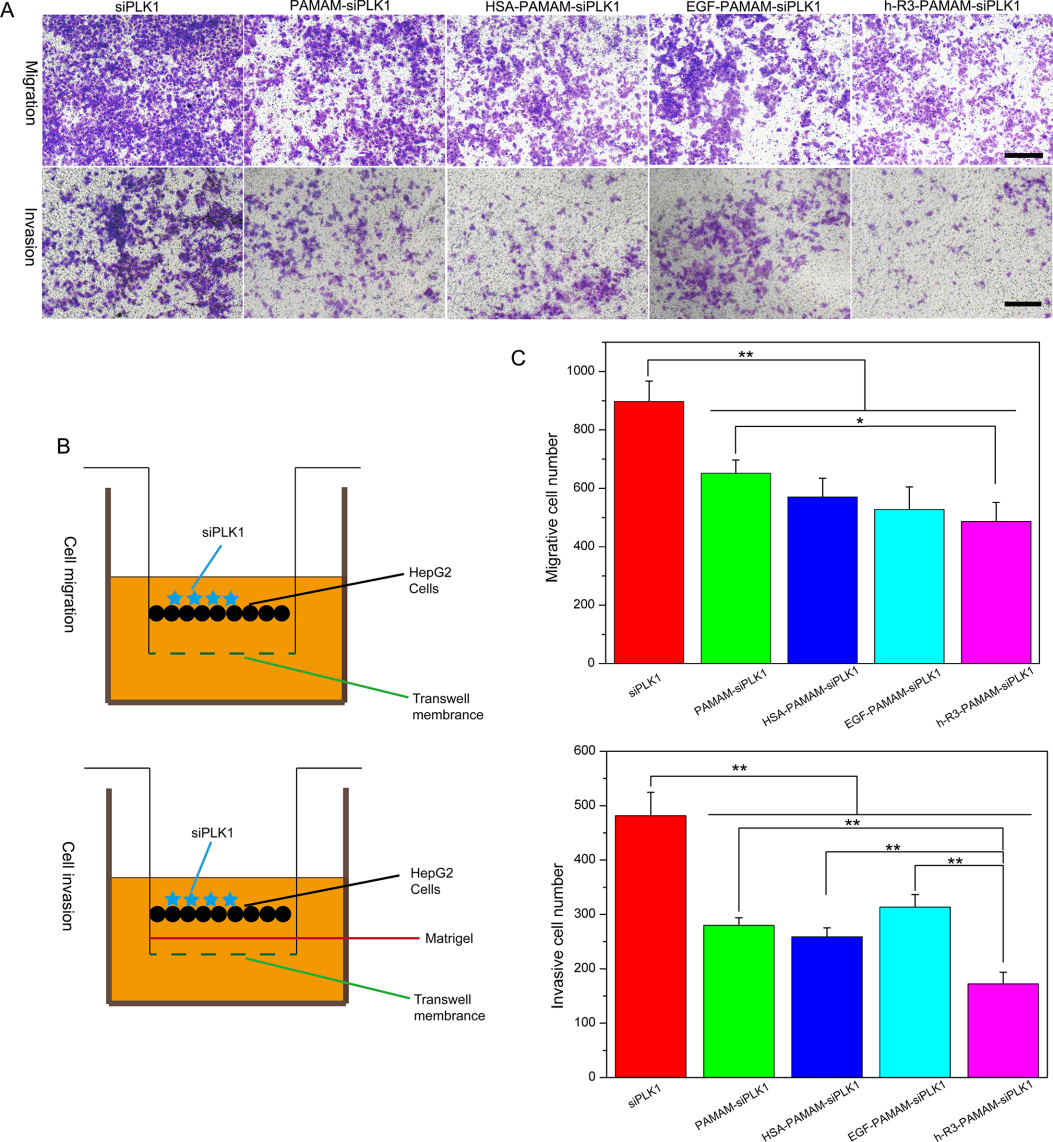
Inhibition of migration and invasion after transfection (**A**) Significantly decreased cell migration/invasion was observed in HepG2 cells with siPLK1-mediated knockdown. Scale bars = 150 μm. (**B**) Schematic for the cell migration/invasion assays. (**C**) Quantitative analysis of migrative/invasive cell numbers per field was executed by Image-Pro Plus. Error bars indicate s.d. (*n* = 3). *P < 0.05, **P < 0.01.

Cancer cell migration and invasion are two critical initiation steps in the process of tumor metastasis [57]. To further characterize the effect of siPLK1 delivery in HepG2 cells, we investigated the migration and invasion of transfected cells *in vitro* by transwell assays, and the schematic for the cell migration/invasion assays was shown in Figure 9B. The number of cells transfected with h-R3-siPLK1-PAMAM (486.33 ± 65.55) migrating through the chamber was lower than cells transfected with siPLK1 (897.42 ± 70.23), siPLK1-PAMAM (651.21 ± 45.60), HSA-siPLK1-PAMAM (570.62 ± 63.96) and EGF-siPLK1-PAMAM (528.00 ± 76.86) (Figure 9A and 9C). The same result was also observed in parallel invasion assays with h-R3-siPLK1-PAMAM (172.57 ± 21.46), siPLK1 (481.67 ± 42.81), siPLK1-PAMAM (280.02 ± 13.89), HSA-siPLK1-PAMAM (259.00 ± 16.52) and EGF-siPLK1-PAMAM (303.25 ± 23.35) (Figure 9A and 9C). The highly reduced migration and invasion were observed in HepG2 cells after knockdown of PLK1 by transfected with h-R3-siPLK1-PAMAM complexes. This finding shows the potential contributive role of h-R3-siPLK1-PAMAM in the inhibition of tumor metastasis.

## CONCLUSIONS

Dendriplexes and ligand (h-R3/EGF/HSA)-dendriplexes as siRNA delivery carriers were designed through self-assembly in this paper. We characterized the physicochemical properties (including siRNA loading ability, particles size, zeta potential and morphology), evaluated *in vitro* toxicity, gene transfection efficacy and intracellular uptake. Furthermore, we examined the *in vivo* distribution and gene delivery of dendriplexes and ligand-dendriplexes in tumor-bearing BALB/c nude mice. Dendriplexes and ligand-dendriplexes represented excellent siRNA encapsulation ability, formed unique nanostructures and presented lower cytotoxicity. Compared to dendriplexes, HSA-dendriplexes and EGF-dendriplexes, h-R3-dendriplexes showed greater cellular uptake and excellent endosomal escape capacity. Moreover, the in *ex vivo* distribution in the tumors also confirmed that h-R3-dendriplexes had much better gene delivery efficiency and high targeted delivery than dendriplexes, HSA-dendriplexes and EGF-dendriplexes. Overall, the modification of h-R3 with dendriplexes on the surface contributes better efficiency to siRNA delivery *in vitro* and *ex vivo*. Furthermore, h-R3-dendriplexes for siPLK1 delivery represented efficient cell growth inhibition, potentiated cell apoptosis and suppressed cellular migration/invasion. Therefore, we believe that h-R3-dendriplexes represent a promising targeted siRNA delivery candidate.

## MATERIALS AND METHODS

### Materials

Poly(amidoamine) (PAMAM) dendrimer with a ethylenediamine core (generation 5 with 128 surface amino groups) was purchased from Sigma (Shanghai, China). Nimotuzumab (h-R3) was a gift from BioTech Pharmaceuticals Co., Ltd (Beijing, China). Human serum albumin (HSA) and epidermal growth factor (EGF) were purchased from Shandong Taibang Biological Products Co., Ltd (Taian, China) and Sigma (Shanghai, China), respectively. Cy5-labeled siRNA was purchased from Suzhou Ribo Life Science Co., Ltd (Kunshan, China), and the sequences were as follows: sense strand, 5′-UUCUCCGAACGUGUCACGUdTdT-3′; antisense strand, 5′-ACGUGACACGUUCGGAGAAdTdT-3′. Cy5 fluorophore was labeled at 5′ of the sense strand. NC siRNA was supplied by Suzhou Ribo Life Science Co., Ltd, and the sequences were as follows: sense strand, 5′-CGGAAGGCCUAAUGCCGAAdTdT-3′; antisense strand, 5′-UUCGGCAUUAGGCCUUCCGdTdG-3′. And, siPLK1 has the sequences as follows: sense strand, 5′-UGAAGAAGAUCACCCUCCUUAdTdT-3′; antisense strand, 5′-UAAGGAGGGUGAUCUfUCUfUCfAdTdT-3′. The fluorescent dye DAPI was purchase from Fanbo Biochemical Co (Beijing, China). Fluorescein isothiocyanate-labeled phalloidin, 3-(4,5-Dimethylthiazol-2-yl)-2,5-diphenyltetrazolium bromide (MTT) and dimethylsulfoxide (DMSO) were purchased from Sigma (Shanghai, China). All other reagents were obtained from the Biodee Reagent Company (Beijing, China).

### Cell culture

HepG2 cells were obtained from China Center for Typical Culture Collection (Beijing, China) and cultured in Dulbecco's Modified Eagle's Medium (DMEM) (Gibco, USA), supplemented with 10% fetal bovine serum (FBS) and 100 unit/ml penicillin/streptomycin. All cell lines were cultivated in a humidified atmosphere of 5% CO_2_ at 37°C.

### Preparation of dendriplexes and ligand-dendriplexes

PAMAM was diluted to an appropriate concentration in PBS and stored at 4°C until use. PAMAM-siRNA complexes (dendriplexes) were made up by adding the aqueous solution of siRNA to an equal volume of PAMAM in PBS at a particular N/P ratio, followed by incubation for 20 min. The N/P ratio was based on the calculation of the number of terminal -NH2 groups on the dendrimer versus the number of phosphate groups of the siRNA. H-R3/EGF/HSA-dendriplexes were prepared by dropping the dendriplex aqueous solution to an aqueous solution of ligand (h-R3/EGF/HSA) of equal volume and then incubating for 20 min.

### Size distribution and zeta potential measurements

Size distribution and zeta potential of the dendriplexes and h-R3-dendriplexes were measured using a Zetasizer Nano ZS90 (Malvern Instruments Ltd, Malvern, UK). Measurements were performed at a temperature of 25°C after equilibration for 2 min. All results were the mean of three test runs.

### Agarose gel electrophoresis

Dendriplexes and h-R3/EGF/HSA-dendriplexes with different compositions were evaluated by agarose gel retardation assay. Twenty microliters of complexes containing solution with 1 μg DNA was electrophoresed on the 1% (w/v) agarose gel containing ethidium bromide with Tris-acetate-EDTA (TAE) running buffer at 110 V for 30 min. DNA was visualized on a Vilber Lourmat UV transilluminator.

### Transmission electron microscope (TEM)

The dried specimens were examined with a Zeiss EM 900 transmission electron microscope at an acceleration voltage of 80 kV. Electron micrographs were taken with a slow scan camera (Variospeed SSCCD camera SM-1k-120, TRS, Moorenweis, Germany).

### *In vitro* cytotoxicity

The cytotoxicity of dendriplexes and ligand-dendriplexes with different formulations were examined by MTT assay. Cells were seeded in 96-well plates at a density of 10^4^ cells/well in 200 μl of DMEM containing 10% FBS. After incubation for overnight, the dendriplex/h-R3-dendriplex opti-MEM solution (20 μl containing 0.2 μg siRNA) was added into each well. After 4 h, 200 μl fresh complete DMEM was added, and transfection proceeded for an additional 44 h in the presence of 10% FBS. Then, 20 μl of MTT (5 mg/ml) solution was added to each well, and further incubated for 4 h. Thereafter, the medium was carefully removed and 150 μl DMSO was added to each well to dissolve the formazan crystals. The absorbance was measured at 490 nm by a microplate reader. The cells without co-incubation with the dendriplexes were used as the control.

### Cellular uptake

For cellular uptake study, 5 × 10^4^ HepG2 cells were seeded in 24-well culture plates for overnight before transfection. The cells were treated with complexes solution containing 2 μg Cy5-siRNA (N/P 20:1, ligand/siRNA 0.5) was added into each plate. After 5 h incubation at 37°C in 5% CO_2_ humidified atmosphere, the transfection solutions were aspirated and substituted with complete culture medium, and the cells were further incubated for 24 hours in medium containing 10% FBS. After transfection, cells were detached by trypsin. Cell suspensions were then transferred to microtubes and fixed by 0.2 mM EDTA. The percentage of cells transfected was quantitated by flow cytometry using fluorescence-activated cell sorting (FACS) machine.

### Confocal laser scanning microscopy (CLSM)

HepG2 cells were plated at 10^5^ cells/well 12 h before transfection. After the culture medium was changed to opti-MEM, well incubated complexes solution containing 2 μg Cy5-siRNA (N/P 20:1, ligand/siRNA 0.5) was added into each plate. After 5 h incubation at 37°C in 5% CO_2_ humidified atmosphere, the transfection solutions were aspirated and substituted with complete culture medium. After additional 19 h incubation, the cells were washed with PBS for three times before acidic late endosomes staining with LysoTracker green (Life Technologies). For confocal laser scanning microscopic measurements, a laser scanning microscope LSM 710 with Plan-Apochromat 100×/1.40 Oil DIC M27 objective (Zeiss, Germany) was used.

### *Ex vivo* distribution

All procedures of the *ex vivo* experiments complied with the standards for use of animal subjects as stated in the guidelines from Committee on Animal Research in Tsinghua University. The *ex vivo* studies were performed in female BALB/c nude mice from the Experimental Animal Center (Tsinghua University, Beijing, China). They were housed under controlled conditions (12 h light/dark schedule, 24°C) in groups of three mice per cage and formulation. Tumors were introduced in the armpits of the nude mice by inoculation with 10^7^ HepG2 cells. Palpable subcutaneous tumors developed over a period of 14 days. To monitor dendriplex, h-R3-dendriplex, EGF-dendriplex and HSA-dendriplex *in vivo*, Cy5-siRNA was used. Distribution was assessed by injecting dendriplexes and h-R3/EGF/HSA-dendriplexes (N/P 20:1, ligand/siRNA 0.5) into tumor-bearing mice via the tail vein in a total volume of 200 μl. The mice were sacrificed and organs were subjected for *ex vivo* imaging by the Kodak *in vivo* imaging system FX-Pro. The optical imaging was obtained using Kodak multimodal imaging system FX-Pro equipped with an excitation bandpass filter at 630 nm and an emission at 700 nm. Region-of-interests were circled around the organs, and the fluorescence intensities were analysed with the Carestream MI SE 5.4.2 software package.

### Cryosection preparation and *ex vivo* confocal observation

The tumor tissues were placed on omnisette tissue cassettes and frozen rapidly to −20°C. At this temperature, the tissues become rock-hard. The specimens were embedded in optimal cutting temperature compound, and cut into 6 mm histology slices by the cryostat. Each section was picked up on glass slides, stained by DAPI (staining nuclei) and Phalloidin (staining F-actin, which can display the rough cell outline), and then covered with coverslip. Finally, cryosections were observed using a confocal microscope (LSM 710, Zeiss, Germany).

### RT-PCR for PLK1 mRNA detection

HepG2 cells were plated in a 6-well plates (4 × 10^5^ cells per well) and transfected with different samples containing 2 μg siPLK1 (naked siPLK1, PAMAM-siPLK1, HSA/EGF/h-R3-PAMAM-siPLK1) for 48 h. Total RNA was isolated with TransZolTM Up (TransGen Biotech, Beijing, China). The reverse transcription reaction was performed using TransScript First-Strand cDNA Synthesis SuperMix System (TransGen Biotech, Beijing, China). For detection of PLK1 transcript, 3 μl cDNA was used in 20 μl reaction with PLK1 primers. A volume of 20 μl PCR reaction was electrophoresed on a 1% agarose gel, and the amplified DNA band was visualized by DuRed (Bridgen Biotech, Beijing, China) staining.

### MTT assay for studies of cell growth curve and therapy efficacy

HepG2 cells were seeded in 96-well plates at a density of 10^4^ cells/well in 200 μl of DMEM containing 10% FBS. After incubation for overnight, the samples with different siPLK1 concentration (2 μg/ml, 5 μg/ml, 10 μg/ml) were added into each well for transfection. Then, after 6 h, 200 μl fresh complete DMEM was added, and transfection proceeded for an additional 18 h or 42 h in the presence of 10% FBS. Then, the MTT detection method was described as *in vitro* cytotoxicity part.

### Apoptosis analysis by annexin V-FITC and propidium iodide (PI) double staining

HepG2 cells were plated in a 6-well plates (4 × 10^5^ cells per well) and transfected as described above. After incubation with treated samples for 48 h, the treated cells were washed, trypsinized and centrifuged. The cells were collected and re-suspended in 200 μl of Binding buffer, and 10 μl Annexin V-FITC and 10 μl PI (Solarbio LIFE SCIENCES, Beijing, China) were added. Furthermore, the stained cells were incubated at room temperature for 15 min in the dark, and analyzed by FACS Calibur flow cytometer with WinMDI 2.9 software.

### Cell migration and invasion assay

Cell migration and invasion was measured using transwell chamber (8 μm, 24-well format; Corning, Lowell, MA, USA). To measure migration, 2 × 10^5^ HepG2 cells after transfection with different samples containing 1 μg siPLK1 (naked siPLK1, PAMAM-siPLK1 and HSA/EGF/h-R3-PAMAM-siPLK1) were resuspended in 0.2 ml of serum-free medium and added to the upper chamber, and 0.5 ml of medium containing 10% FBS was added to the lower chamber. Cells were incubated for 24 hours. To measure invasion, 60 μl diluted Matrigel (BD Biosciences, MD, USA) was used to coat the insert chambers' membrane. Cells were cultured for 24 h under the same conditions. Finally, cells that migrated or invaded into the lower chambers were fixed with methanol, stained with crystal violet. The number of cells was counted using an Olympus IX73 research microscope (Olympus, Tokyo, Japan)

### Statistical analysis

Statistical significance between two samples was performed using two-tailed Student's *t*-tests. When multiple samples were compared, statistical significance was assessed using one-way ANOVA followed by Dunnett's-test. A value of *P* < 0.05 was considered as statistically significant, and *P*-value < 0.01 was considered as highly significant.
